# A comparative evaluation of microleakage and dentin shear bond strength of three restorative materials

**DOI:** 10.1080/26415275.2022.2033623

**Published:** 2022-02-10

**Authors:** Alpana Kumari, Namita Singh

**Affiliations:** Department of Pedodontics and Preventive Dentistry, Christian Dental College, Ludhiana, CMC, Ludhiana, India

**Keywords:** Microleakage, shear bond strength, glass ionomer cement, Zirconomer, alkasite

## Abstract

**Aim:**

To evaluate the microleakage and dentin shear bond strength of two glass containing restorative materials, Zirconomer and Cention N, and to compare them with a conventional glass ionomer cement (GIC) (GC Fuji II).

**Materials and methods:**

Zirconomer (Shofu) and GC Fuji II (GC Corp.) are self-curing GICs whereas Cention N (IvoclarVivadent) also offers a self-curing option as well as the option of light-curing using an adhesive. For evaluating microleakage, standardized class V cavities were prepared on the buccal surface of 30 premolars. The cavities were restored with one of the three restorative materials (*n* = 10) according to manufacturers’ instructions, Cention N being used with an adhesive (Te-EconomBond, IvoclarVivadent) and in the light-curing mode. After restoration and thermocycling, the microleakage assessment was made under a stereomicroscope at 40x magnification following immersing of the teeth in 0.5% methylene blue dye and buccolingual sectioning. For evaluating dentin shear bond strength, the occlusal surface of the 30 premolars was ground flat, and cylinders of the three restorative materials (*n* = 10) were bonded to the occlusal surface according to manufacturers’ instructions, Cention N being used with an adhesive (Te-EconomBond, IvoclarVivadent) and in the light-curing mode. Following 24-h storage at 100% humidity, the dentin shear bond strength was measured and the fracture mode was determined under a stereomicroscope at 10× magnification. Data were statistically analyzed using Mann–Whitney and Scheffé tests (*p* = .05).

**Results:**

Cention N displayed significantly less microleakage than did Zirconomer and GC Fuji II at occlusal as well as the gingival margins. Dentin shear bond strength varied significantly between 5.15 and 9.89 MPa with Cention N showing the highest bond strength and GC Fuji II the lowest.

**Conclusion:**

In this *in vitro* evaluation, Cention N consistently performed better than the conventional GIC (GC Fuji II) as well as Zirconomer.

## Introduction

The human tooth has a limited capacity for regeneration. Therefore, replacing the lost tooth structure becomes imperative to maintain the tooth form, its function, esthetics, and clinical longevity [1]. Over the years, studies have shown that conventional restorative materials and techniques fail to provide a complete marginal seal with the tooth resulting in the leakage of fluid and consequently causing post-operative sensitivity, marginal discoloration, impaired marginal integrity, and secondary caries [2,3]. Furthermore, the clinical effectiveness of newer restorative material is based upon strong adhesion with the dentinal surface to combat various dislodging forces acting on the tooth [4].

Glass ionomer cement (GIC) has been successfully used as dental restorative material following its invention by Wilson and Kent in the early 1970s [5]. The unique properties of GIC's are their adhesion to moist tooth surfaces, anti-cariogenic character, lack of exothermic polymerization, excellent adhesion to dentin, and satisfactory biocompatibility [6]. One of the major drawbacks of GICs is their weak mechanical properties like brittleness, low strength, and toughness [5,6]. Because of their poor mechanical strength, GICs were mainly used to restore anterior teeth and in areas with minimal load [6].

A new generation of GICs called Zirconomer has been developed by Shofu Inc., Japan, that intends to overcome the drawbacks of previously used tooth-colored restorative materials [6]. The structural integrity of restorations is reinforced due to the inclusion of zirconia fillers in the glass component of Zirconomer and hence imparting superior mechanical properties in posterior load-bearing areas [6,7].

Another type of glass containing posterior, direct filling, tooth-colored, restorative material named Cention N (IvoclarVivadent, Liechtenstein) has been introduced as a ‘powder-liquid filling material’. It is a urethane dimethacrylate alkasite restorative material that utilizes alkaline filler and releases acid-neutralizing ions [8]. The presence of isofiller having a low modulus of elasticity supposedly acts as a shrinkage stress reliever, thus reducing microleakage and polymerization shrinkage. As it contains alkaline glass fillers, it is also capable of releasing fluorides, calcium, and hydroxide ions, which have beneficial effects, especially in the pediatric scenario [8,9]. Being dual-cured this material is used for bulk placement with or without the use of adhesives. As per the literature, using adhesive increases the sealing ability of Cention N [10].

Only a limited number of studies have been conducted to compare the *in vitro* performance of newer modified restorative materials. Therefore, the present study was undertaken to evaluate and compare two important aspects, microleakage, and dentin shear bond strength, of Zirconomer and Cention N with that of GC Fuji II, a conventional glass ionomer cement. The null hypothesis of the present study was that there is no difference in the microleakage and dentin shear bond strength between the three restorative materials tested.

## Materials and methods

After obtaining clearance and approval from the concerned authorities (BFUHS/2k19/p.TH/13234), a total of 60 sound premolar teeth extracted for orthodontic purposes were procured. Each tooth was thoroughly scaled to remove calculus and remaining tissue tags and then polished with a pumice slurry. The teeth were stored in saline until use [11]. The restorative materials used are listed in Tables 1 and 2.

**Table 1.** Armamentarium.

Cention N	IvoclarVivadent, Schaan, Liechtenstein (LOT: Liquid: Y27738, Powder: Y24910)
Zirconomer	Shofu Inc., Kyoto, Japan (LOT: 07181280)
GC Fuji II	GC Corporation, Tokyo, Japan (LOT: 1909051)
Eco-Etch	IvoclarVivadent, Schaan, Liechtenstein (LOT: Y33753)
Te-EconomBond	IvoclarVivadent, Schaan, Liechtenstein (LOT: Y28755)
Woodpecker visible light curing unit	Zhengzhou Linker Medical Equipment Co., Henan, China
Nail varnish	COLORBAR, USA
Stereomicroscope	GOKO MIAMB ISO 9001:2008, Department of Oral Pathology and Microbiology, Christian Dental College, Ludhiana, India
Universal testing machine	Capacity: 2.5 KN, Model: WDW, Make: JINAN
Micromotor	Marathon 600) With Straight Handpiece
Diamond disc	No. 1219218, DFS, Germany

**Table 2.** Composition of the restorative materials.

Material	Composition
Cention N	Liquid
	Dimethacrylate
	Initiators
	Stabilizers
	Additives
	Mint flavour
	Powder
	Calcium fluoro silicate
	Glass
	Barium glass
	Calcium barium aluminium
	Fluoro silicate glass
	Iso fillers
	Ytterbium tri
	Fluoride
	Initiator
	Pigments
Zirconomer	Liquid
	Polyacrylic acid solution
	Tartaric acid
	Powder
	Fluoroaluminosilicate
	Glass
	Zirconium oxide
	Pigments and others
GC Fuji II	Liquid
	Polyacrylic acid
	Itaconic acid
	Maleic acid
	Tricarballylic acid
	Tartaric acid
	Water
	Powder
	Silica
	Alumina
	Aluminium fluoride
	Calcium fluoride
	Sodium fluoride
	Aluminium phosphate
	Lanthanum, strontium
	Barium in traces

### Microleakage

**Figure 1.**
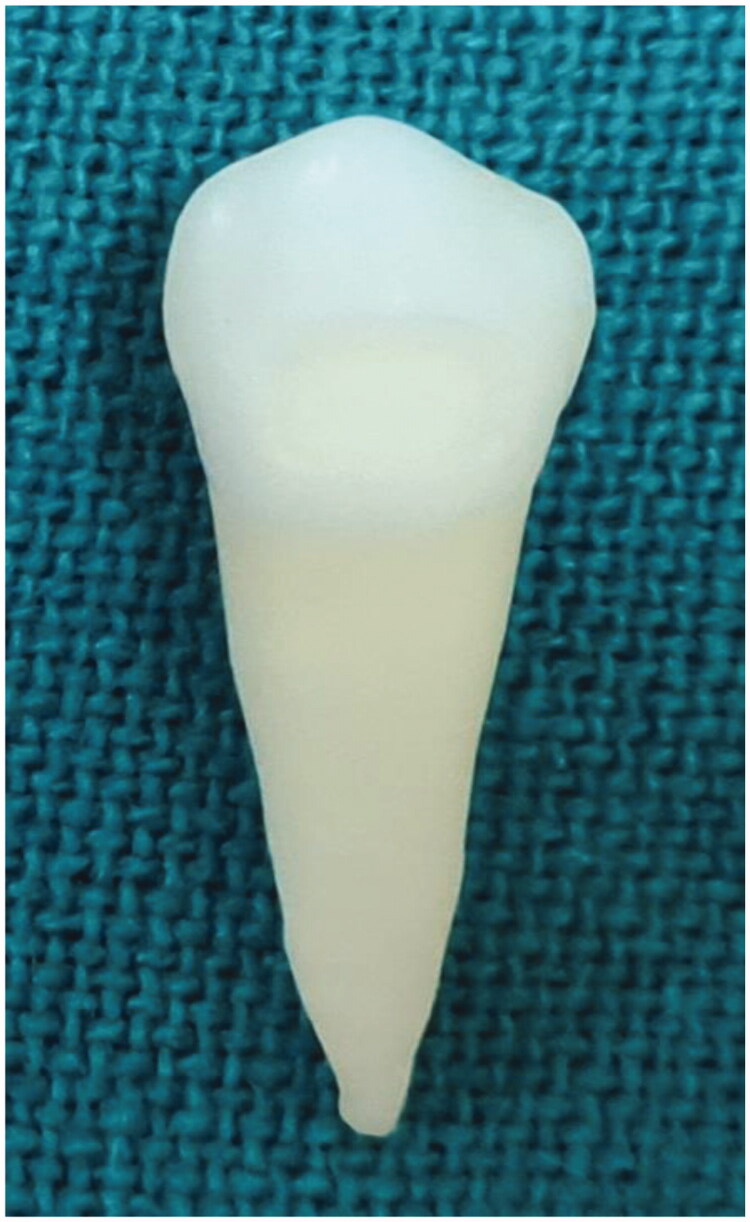
0 = No dye penetration,1 = dye penetration along the interface to one-third of the cavity depth, 2 = dye penetration along the interface to two-thirds of the cavity wall depth, 3 = dye penetration up to, but not along, the axial wall, and 4 = dye penetration up to and along the axial wall.

Class V cavities were prepared on the buccal surface of 30 premolars using a No. 1 round bur and high-speed air rotor with water spray. Gingivally, the cavity margin was placed 1 mm above the cementoenamel junction (CEJ). The cavity dimensions were 3 mm in length, 2 mm in width, and 1.5 mm in depth (Figure 1). The cavities were standardized using a divider, digital caliper, and a graduated probe to validate the cavity's depth. The prepared teeth were randomly divided into three groups of 10.

#### Cavities restored with GC Fuji II

The prepared cavity was rinsed thoroughly with air/water spray and dried. GC Fuji II was mixed according to the manufacturer's instructions (powder: liquid 2.7:1 part by weight) and was placed into the cavity using a plastic filling instrument. The setting time of GC Fuji II is 2 min 20 s. The restoration was finished using finishing burs after waiting for 5 min and petroleum jelly was applied.

#### Cavities restored with Zirconomer

The prepared cavity was rinsed thoroughly with air/water spray and dried. Zirconomer was mixed according to the manufacturer's instructions (powder: liquid 3.6:1 part by weight) and was placed into the cavity using a plastic filling instrument. The setting time of Zirconomer is 3 min. The restoration was finished using finishing burs after waiting for 5 min and petroleum jelly was applied.

#### Cavities restored with Cention N

The prepared cavity was rinsed thoroughly with air/water spray and dried. Etching (Eco-Etch, IvoclarVivadent, Liechtenstein) and bonding (Te-EconomBond, IvoclarVivadent) of cavity surfaces were done for 15 and 10 s, respectively. Subsequently, Cention N (IvoclarVivadent) cement was mixed according to manufacturer’s instructions (powder:liquid 4.6:1 part by weight) and placed into the cavity using a plastic filling instrument and light-cured with a visible light curing unit (Woodpecker) for 20 s and then immediately finished and polished using burs.

The restored teeth after the application of petroleum jelly were left undisturbed for 24 h at room temperature and at ambient humidity, after that, they were stored in distilled water at room temperature for 24 h before thermocycling. The thermocycling process was done according to the ISO: 11405 protocol [12]. All restored teeth were subjected to thermocycling at 5 and 55 °C for 500 cycles with 5 s immersion time in each water bath.

The root apices were then sealed with sticky wax, and the tooth surfaces except for the restoration and a 1 mm zone adjacent to its margin were covered with two coats of nail varnish. Subsequently, the specimens were immersed in 0.5% methylene blue dye for 24 h and then rinsed thoroughly with water to remove the residual dye. The teeth were then embedded in acrylic blocks and sectioned buccolingually in the approximate centre of the restoration using a micromotor with a diamond disc. The degree of microleakage of both halves of the restored teeth was assessed at the gingival and the occlusal margin using a stereomicroscope under 40x magnification and the following scoring criteria [13].

0: No dye penetration
1: Dye penetration along with the interface to one-third of the cavity depth
2: Dye penetration along with the interface to two-third of the cavity depth
3: Dye penetration up to, but not along the axial wall
4: Dye penetration up to, and along the axial wall

### Dentin shear bond strength

The occlusal surface of the remaining thirty premolars was exposed by a flat cut perpendicular to the longitudinal axis of each tooth using a high-speed diamond disc and copious water spray. This was followed by polishing the dentin surface with 600 grit silicon carbide paper. The specimens were stored in distilled water at room temperature until mounting of the roots in self-polymerizing acrylic resin using silicone moulds of 25*25 mm dimensions with the coronal surfaces protruding from the acrylic blocks. The embedded teeth were then randomly divided into three groups of 10. Double-sided adhesive tape with a punch hole of diameter 3 mm was applied on the flat dentin surface of each tooth specimen to delineate an area for bonding.

A plastic mould measuring 3 mm in internal diameter and 5 mm in height was placed in the center of the exposed dentin surface. Each restorative material was mixed as described above and filled into the cylinder using a plastic spatula. The setting time of GC Fuji II is 2 min 20 s, Zirconomer is 3 min and self-cured Cention N is ≤5 min as per the manufacturer but since the Cention N was light-cured with visible light curing (Woodpecker) for 20 s, it was immediately set. Once the set of restorative material was confirmed after 6–7 min, the cylinder was removed carefully by making two parallel cuts with a Bard Parker blade without breaking the restoration.

The specimens were stored at 37 °C and 100% humidity in an incubator for 24 h [14]. Each specimen was then mounted in a universal testing machine (JINAN, China) with the dentin surface kept parallel to the machine. The load was applied using a steel knife edge placed at the dentin-restoration interface. The shear force was applied directly to the interface at a crosshead speed of 0.5 mm/min until restoration failure occurred.

The force (N) required to displace the restoration was recorded, and the bond strength (MPa) was calculated by dividing the shear force by the bonding area (mm^2^). After the shear test, the tooth specimens were examined under a stereomicroscope at 10× magnification for fracture mode analysis.

Fracture modes were classified as [15]:

**Adhesive** (failure at the interface between restoration and dentin)

**Cohesive** (failure within the restorative material)

**Mixed** (partly adhesive and partly cohesive fracture)

A single trained investigator performed all the procedures.

### Statistical analysis

The microleakage and bond strength results were tabulated and statistically analyzed using SPSS (Statistical Packages for Social Sciences, version 21.0., IBM Corp., Armonk, NY, USA). The microleakage results were analyzed using Mann-Whitney tests while the bond strength results were analyzed using *post-hoc* Scheffe and ANOVA tests. The level of significance was set at *p* = .05.

## Results

### Microleakage

**Figure 2.**
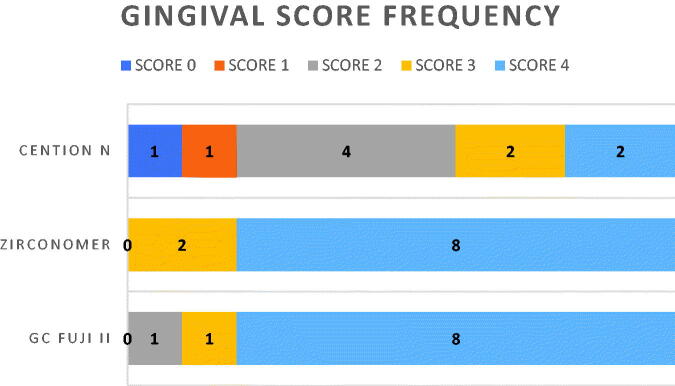
Gingival score frequency. 0 = No dye penetration, 1 = dye penetration along the interface to one-third of the cavity depth, 2 = dye penetration along the interface to two-thirds of the cavity wall depth, 3 = dye penetration up to, but not along, the axial wall and 4 = dye penetration up to and along the axial wall.

**Figure 3.**
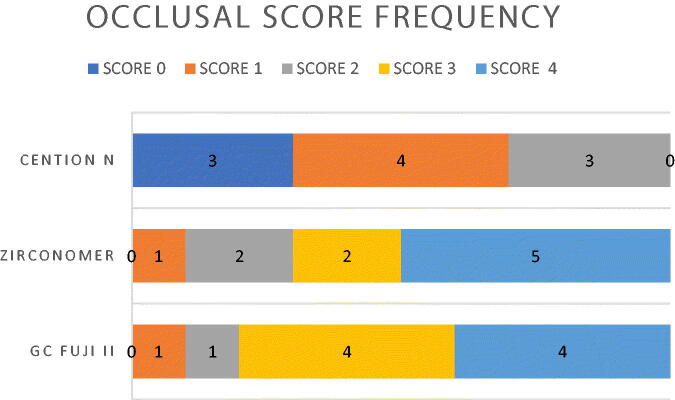
Occlusal score frequency.

Gingival and occlusal microleakage scores of the three-restorative materials are presented in Figures 2, 3. As shown in Table 3, Cention N restorations displayed significantly less microleakage than did restorations of either Zirconomer or GC Fuji II at the gingival as well as occlusal margins. No significant differences were found between the latter two materials. Whereas Cention N restorations displayed less microleakage at the occlusal margin than at the gingival margin (Table 4), no significant differences were found between the two locations for Zirconomer and GC Fuji II, respectively.

**Table 3.** Pair-wise comparison of microleakage results.

	Gingival score		Occlusal score	
	*Z* score	*p*-Value	*Z* score	*p*-Value
GC Fuji II *vs.* Zirconomer	−0.038	.968	−0.076	.936
GC Fuji II *vs.* Cention N	2.457	.014*	3.251	.001*
Zirconomer *vs.* Cention N	2.683	.007*	3.137	.002*

**Table 4.** Comparison of gingival and occlusal scores.

Comparison of gingival and occlusal scores	*Z* scores	*p*-Value
GC Fuji II Gingival *vs.* Occlusal	1.436	.150
Zirconomer Gingival *vs.* Occlusal	1.323	.187
Cention N Gingival *vs.* Occlusal	2.268	.023*

### Dentin shear bond strength

The bond strength to dentin varied between 5.15 and 9.89 MPa (Table 5). As shown in Tables 6, 7, statistically significant differences were found between all three materials. Thus, GC Fuji II had the significantly lowest bond strength, followed by Zirconomer and with Cention N having the significantly highest bond strength.

**Table 5.** Dentin shear bond strength (MPa).

Group	Number of samples	Mean	Standard deviation
GC Fuji II	10	5.15	1.22
Zirconomer	10	7.48	1.02
Cention N	10	9.89	1.23
Total	30	7.51	2.27

**Table 6.** ANOVA analysis of shear bond strength.

Source of variation	Sum of squares	*df*	Mean square	*F*	*p*-Value
Between groups	112.4891	2	56.24456	41.88306	<.001
Within groups	36.25817	27	1.342895		
Total	148.7473	29			

**Table 7.** Multiple comparison of dentin shear bond strength using *post-hoc* Scheffe’s test.

Group	Group	Mean difference	Std. error	Sig.	95% Confidence interval	
					Lower bound	Upper bound
GC Fuji II	Zirconomer	−2.335	0.518	0.001	−3.677	−0.993
	Cention N	−4.743	0.518	0.000	−6.085	−3.401
Zirconomer	GC Fuji II	2.335	0.518	0.001	0.993	3.677
	Cention N	−2.408	0.518	0.000	−3.750	−1.066
Cention N	GC Fuji II	4.743	0.518	0.000	3.401	6.085
	Zirconomer	2.408	0.518	0.000	1.066	3.750

**Figure 4.**
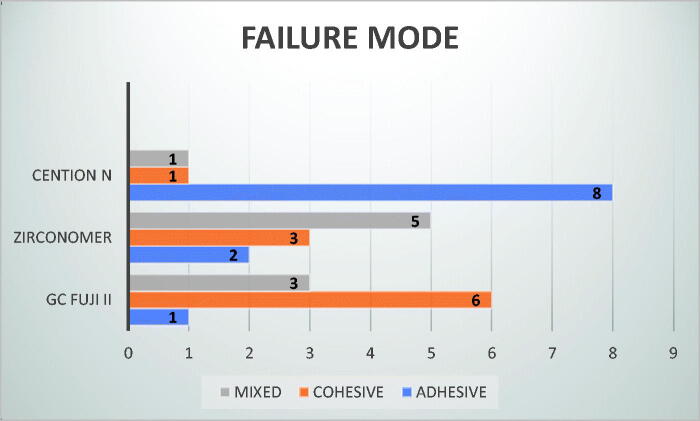
Failure mode.

According to the failure mode analysis, GC Fuji II resulted mainly in cohesive failures, Zirconomer mainly in mixed failures, whereas Cention N resulted predominantly in adhesive failures (Figure 4).

## Discussion

The present study evaluated the microleakage and dentin bond strength of two modified restorative materials, Zirconomer and Cention N, and compared them with the performance of GC Fuji II, a conventional glass ionomer cement. The null hypothesis of the study was rejected as there were differences in the microleakage and in dentin bond strength between the three restorative materials.

Modern dentistry stands on the parapet of preserving and protecting the integrity of dental hard tissue [16]. According to the literature, the clinical success of any restorative material depends mainly on its excellent adhesion with the dentinal surface to resist various dislodging forces bestowed upon them in the oral cavity. Further, this adhesion with the tooth surface preserves the marginal integrity, preventing microleakage [17].

In the present study, the simple and widely used method of 0.5% methylene blue was used to evaluate microleakage after restoring the class V cavities on the buccal surfaces of human premolars. The diameter of the dye molecule is 0.80 nm which is less than the diameter of dentinal tubules [18]. Permeability of dentinal tubules along with the smaller particle size can cause an over estimation of the relevance of this penetration [19]. A similar dye was also used in studies done by Raju et al. [20] and Meshram et al. [21]. Thermocycling was carried out to simulate the thermal changes seen in the oral cavity.

For all the restorative materials under consideration, it was found that the occlusal margin had lower microleakage scores than did the gingival margin. This finding is in accordance with a previously reported *in vitro* study of Bahsi et al. [3].

However, on comparing the gingival and occlusal scores for each material, a statistically significant difference in microleakage was found for Cention N whereas for Zirconomer and GC Fuji II it was statistically insignificant. Less microleakage at the occlusal margin could be due to the fact that the enamel adhesion is stronger than the dentin adhesion and/or that enamel has a higher mineral content [22]. Nevertheless, Mazaheri et al. [23] concluded in their study that the microleakage score was not significantly different at occlusal and gingival margins.

In this study, Zirconomer had higher microleakage than Cention N. Patel et al. got similar results when Zirconomer was compared with different materials. The reason for the higher microleakage could be the large size of the filler particles in Zirconomer, which prevents proper adaptation of this material to the tooth surface [24].

The lowest microleakage was shown by the Cention N restorations at both the occlusal and the gingival margins. This superior behavior could be due to the isofiller present in the powder of Cention N, which acts as a shrinkage stress reliever. Allegedly the presence of a unique patented filler is responsible for keeping the shrinkage stress to the minimum. Likewise, the organic/inorganic ratio and the monomer composition of the material are accountable for decreased volumetric shrinkage [9]. Furthermore, the silanes bonded to filler particles improve the bond between the inorganic filler. The result of this study is also similar to that of the study conducted by Naz et al. in which the mean microleakage of Zirconomer was found to be greater than that of Cention N [25].

Another parameter that was investigated in the present *in vitro* study was dentin shear bond strength. The high bond strength of restorative materials allows restorations to resist various dislodging forces acting on them. Furthermore, a restorative material that can endure shear and flexural forces during tooth loading should be used in the cervical area of the teeth [26].

The dentin shear bond strength of Cention N was found to be the highest, followed by that of Zirconomer and finally by that of GC Fuji II. The superior bond strength of Cention N corroborated previous findings of Feiz et al. who reported a significantly higher micro-tensile bond strength of Cention N as compared to Zirconomer [27].

The higher bond strength of Cention N could be due to the presence of a stable self-cure initiator along with a highly cross-linked polymer structure. Furthermore, strength to the Cention N is also rendered by the presence of barium aluminium silicate and calcium aluminium silicate glass filler particles making this material more suitable and long-lasting material in the stress-bearing posterior region [9]. The results also indicate that even though the addition of zirconia filler particles improves the mechanical properties of Zirconomer, it doesn’t improve the moisture sensitivity or the tendency of early bond failure of Zirconomer [28].

In this study, Zirconomer had higher bond strength than did the conventional glass ionomer cement GC Fuji II. This result is in accordance with the result of Sapkale et al. [28].

A probable reason for the low shear bond strength obtained in conventional GC Fuji II is the fact that conventional GICs are vulnerable during the initial setting phase [1].

As per the results of our study, Figure 4 shows the different failure modes of the three materials under investigation. Cohesive failure was mainly seen for the conventional glass ionomer, a mixed type of failure for Zirconomer, whereas adhesive failure was most common for Cention N. The latter finding is in accordance with that of Feiz et al. [27], indicating a rupture of the bond at the tooth/restoration interface [29].

For GC Fuji II most failures were of the cohesive type, which is consistent with the previous findings of Kimyai et al. [30], and Kaup et al. [31]. This could be due to the lower mechanical properties as well as the presence of multiple air inclusion bodies, which serve as stress concentration points and thereby increase the chance of a cohesive failure [32,33].

Zirconomer mostly showed mixed failures which are in accordance with the study by Meral and Baseren [34]. This may imply that the interfacial bond strength is higher than the inherent strength of the material responsible for causing cohesive and mixed failure [35].

As the present study was an *in vitro* study with a small sample size, it might not precisely reflect the scenario of the oral environment. Thus, further studies need to be done considering increased sample size and *in vivo* conditions.

## Conclusion

Within the confines of the present *in vitro* study, it can be concluded that Cention N consistently performed better than the conventional GIC and Zirconomer.
